# Dietary glycemic and insulin indices with the risk of osteoporosis: results from the Iranian teachers cohort study

**DOI:** 10.3389/fnut.2024.1415817

**Published:** 2025-01-07

**Authors:** Anahita HoushiarRad, Danial Fotros, Mina Esmaili, Mohammad Hassan Sohouli, Marjan Ajami, Morteza Abdollahi, Motahare Hatami Marbini

**Affiliations:** ^1^Department of Clinical Nutrition and Dietetics, Faculty of Nutrition and Food Technology, National Nutrition and Food Technology Research Institute, Shahid Beheshti University of Medical Sciences, Tehran, Iran; ^2^Student Research Committee, Department of Clinical Nutrition and Dietetics, Faculty of Nutrition and Food Technology, Shahid Beheshti University of Medical Sciences, Tehran, Iran

**Keywords:** osteoporosis, glycemic index, insulin index, glycemic load, insulin load

## Abstract

**Background:**

Osteoporosis is a chronic condition characterized by reduced bone strength and an elevated risk of fractures. The influence of diet and glucose metabolism on bone health and the development of osteoporosis has been an area of interest. This study aimed to investigate the potential association between dietary glycemic index (DGI), dietary glycemic load (DGL), dietary insulin index (DII), dietary insulin load (DIL), and the odds of osteoporosis among Iranian adults.

**Methods:**

Data from 12,696 Iranian teachers (35–50 years) in a cross-sectional study on diet, nutrition, physical activity, and diseases were analyzed. The participants had no history of diabetes, cardiovascular diseases, stroke, thrombosis, or cancer and consumed between 800 and 4,200 kcal/day. We estimated DGI, DGL, DII, and DIL from a validated semi-quantitative food-frequency questionnaire (FFQ). We also diagnosed osteoporosis using dual-energy X-ray absorptiometry.

**Results:**

In the fully adjusted model, higher DGI and DGL were significantly associated with increased odds of osteoporosis (OR = 1.78 and 1.46 for the highest vs. the lowest tertile; *P* trend < 0.05). Nonetheless, no significant association was found between DII or DIL and osteoporosis prevalence. Moreover, higher DIL and DGL were associated with a higher intake of calorie-dense/nutrient-poor foods and a lower intake of antioxidant-rich foods.

**Conclusion:**

Although our study showed that high DGI/DGL increased osteoporosis risk in Iranian teachers, no association was found between DII/DIL and osteoporosis prevalence. More research is needed to confirm these results and understand the mechanisms involved.

## Introduction

Osteoporosis is a chronic and prevalent disease that harms bone strength, leading to increased susceptibility to fractures, impaired physical mobility, and a diminished quality of life (1, 2). More than 200 million people around the world suffer from this disease (3), and its prevalence in Iran, according to the latest statistics, is ~17% (4).

Various factors, such as aging, genetics, certain diseases, some medications, and physical activity and lifestyle, are involved in increasing the risk of osteoporosis (5). Diet is one of the factors that has recently been considered because it can be modified and can play an important role in the prevention, management, and support of drug treatment in these patients (6). As an example, diets and some nutrients such as calcium, magnesium, vitamin D, and vitamin K have been shown to play an important role in bone health and reduce the risk of osteoporosis (7, 8). Moreover, there is a growing body of evidence indicating that diabetes, whether controlled or uncontrolled, may negatively influence bone mineral density (BMD) (9, 10). There are several factors involved in the pathophysiology of bone health regarding hyperglycemia (9–11). In fact, high blood sugar may cause an increase in bone resorption (12). Indeed, the risk of fragility fractures is increased in both patients with type 1 (T1DM) and type 2 (T2DM) diabetes, characterized by chronic hyperglycemia (13–15). However, fragility fractures may occur even in the presence of normal or even slightly elevated BMD in T2DM patients, and the pathophysiological mechanisms of DM-induced skeletal fragility are much more complex, including for instance increased oxidative stress, chronic inflammation, adipokine alterations, and accumulation of advanced glycation end products (13–15). Diets that have a high dietary glycemic index (DGI) and load (DGL), as well as a high dietary insulin index (DII), and load (DIL) cause significant increases in blood glucose and insulin levels (16, 17). Lower glycemic and insulin indexes can reduce inflammation, which may underlie osteoporosis progression (18–20). In general, DGI and DGL are dietary concepts that have been considered to reflect abnormal glucose metabolism and hyperglycemia (21), and DII is a new food ranking algorithm based on the insulin response to the use of isoenergetic reference food in healthy individuals (22). Several studies have demonstrated the existence of a positive correlation between osteoporosis and glycemic and insulin indices in specific populations (23, 24). However, it is crucial to acknowledge the scarcity of research about the connection between DGI, DGL, DII, and DIL and the risk of osteoporosis. This scarcity may hinder the ability to draw definitive conclusions.

Thus, we aimed to investigate the association of glycemic and insulin indices with the odds of osteoporosis in a large sample of the Iranian adult population.

## Methods

### Study participants

The participants investigated in the present study include all male and female teachers aged 35–50 in Iran who are willing to cooperate with the project and have completed the study questionnaires, including the consent questionnaire. The reason for choosing teachers as the investigated sample is as follows: 1. Since the questionnaire is self-administered, the participants must be literate. 2. Due to their job, teachers are fully familiar with how to complete a questionnaire and can understand the concept of questions and filling in the blanks or marking specific places. 3. Teachers are hired and organized by the Ministry of Education, so information about them can be obtained through that ministry. 4. All teachers are insured by the Health Services Insurance Organization and in case of suffering from a serious chronic disease, they will use their insurance. Therefore, if a participant gets sick and does not report it himself, it is possible to get information about his illness by using the database of the health service organization. This cross-sectional study was started in 2001 to determine the relationship between food intake, nutritional status, and physical activity with the incidence of non-communicable diseases. The details related to this study have been reported previously (25). Among the 14,058 participants who entered the present study as primary data, 1,362 people were excluded from the present study due to diabetes, cardiovascular diseases (CVDs), stroke, thrombosis, and cancer, as well as people receiving daily energy outside the range of 800–4,200 and pregnant and lactating women. The data from 12,696 participants were analyzed. In order to determine the sample size in a prospective study to estimate the relative risk, the minimum sample size for estimating with 95% confidence and estimating the relative risk is 1.5, while our estimate is at most 20% away from the actual value of the relative risk and the annual incidence of the desired disease in the non-exposed group is not less than one thousandth (1% during 10 years of study), we need 12,705 samples in each group for comparison. The physical activity levels of the participants were estimated by using a validated short form of the International Physical Activity Questionnaire (Short IPAQ) and reported as the metabolic equivalent of task (MET)-minutes/week (26). Data collection was performed by self-administered questionnaires (23).

### Definition of osteoporosis

The researchers used dual-energy x-ray absorptiometry (GE Healthcare, Madison, WI, USA) with Hardware: Expert and Software: 1.91 to assess the BMD in the study participants. In postmenopausal women and men over 50 years of age in this study, osteoporosis was operationally defined as femoral BMD values that fell 2.5 standard deviations (*T*-scores of −2.5) or below the mean BMD values of Iranian individuals of the same sex within the age range of 20–29 years. The selection of this age group as the reference group is based on the fact that bone mass often reaches its maximum level within this particular range of ages (27). In pre-menopausal women and men under 50 years of age, BMD was expressed as Z-score, and individuals with *Z*-score ≤ -2.0 SD were defined as having BMD “below the expected range for age” (28). For the study purpose, patients with osteoporosis or BMD “below the expected range for age” were classified together as having “low BMD/osteoporosis” (29). Osteopenia is operationally defined as T scores of −1.0 or lower (2, 30). Furthermore, in this study, all secondary causes of osteoporosis were adjusted based on statistical analysis. In fact, all comorbidities, including chronic diseases as well as diseases related to the malabsorption of micronutrients that can affect osteoporosis, have been adjusted based on statistical analysis and their effects have been removed.

### Dietary assessment

The dietary intake over the previous year was obtained using a semi-quantitative food-frequency questionnaire (FFQ) which was specifically developed for this study by experienced experts on food consumption in Iran (25, 31). The FFQ consisted of a list of usual Iranian dietary items with standard serving sizes. For each food item, the average portion size consumed and the frequency of intake were obtained from self-reports on the FFQ. The frequency of intake for each food item included: never, 2–3 times/month, 1 time/week, 2–4 times/week, 5–6 times/week, and daily. The portion sizes were reported in grams using standard Iranian household measures (32). The daily nutrient consumptions for each person were estimated by applying the United States Department of Agriculture's (USDA) national nutrient databank. The Nutritionist IV software (First Databank, San Bruno, CA, USA—modified for Iranian foods) was used to calculate the daily energy and nutrient intake for each participant.

### Calculation of dietary insulin index and load (DII and DIL)

DII for foods containing calories refers to the incremental insulin area under the curve over 2 h in response to the consumption of a 1,000-kJ portion of the test food divided by the area under the curve after ingestion of a 1,000-kJ portion of the reference food. The DII for each calorie-containing food was obtained from FFQ data using data published by Professor Jennie Brand-Miller of the University of Sydney, Australia (33). For each study participant, the total DIL (DILoveral) over the past year, for each calorie-reported food in the FFQ, was determined by calculating its index DII, the calorie content of that food (kcal per portion of that nutrient intake), and its frequency of use (daily portion) and then the sum of the amounts. So, DIL is equal to the summation of (the insulin index of each food x energy content of a serving x number of servings/day of that food). The overall DII (DIIoveral) was also calculated by dividing the DILoveral by total energy intake (kcal/day).

### Glycemic index and glycemic load measurement

The total DGI was calculated using the following formula:

∑ (GI × available carbohydrate)/total available carbohydrate, where the available carbohydrate was calculated as the total carbohydrate minus fiber (34).

The total carbohydrate and fiber contents of the foods were derived from the United States Department of Agriculture food composition table. Of the food and beverage items included in the FFQ, 30 items (17.8%) contained no available carbohydrate. The calculation of the DGL and DGI was thus based on the remaining 138 items, with DGI values ranging from 10 to 123. We used several international (35) and Iranian DGI tables (36) that were previously published. All derived DGI values were relative to glucose as the reference food. The DGI of composite mixed meals was estimated based on the DGI of the individual food components (34). The DGL was calculated as (total GI × total available carbohydrate)/100 (34) and expressed as g/d. The r value for the correlation between carbohydrate intakes derived from the FFQ compared with the average of 3-day dietary records was 0.81, which indicated that the FFQ provides a reasonable measure of total carbohydrate intake over a long period of time (34).

### Anthropometric assessment

The anthropometric measurements were obtained via self-report. The body mass index (BMI) was calculated as weight (kg) divided by the square of the height (m^2^).

### Statistical analyses

All statistical analyses were performed using SPSS software (version 19.0; SPSS Inc, Chicago IL). The normality of variables was evaluated by Kolmogorov–Smirnov and histogram tests. In addition, non-parametric statistics, including the Mann–Whitney *U*-test or Kruskal–Wallis test, were used for variables that were not normally distributed.

The mean values of more than two groups were assessed using analysis of variance (ANOVA) for normal distribution variables. Moreover, for comparing categorical variables, the chi-square test was used. Furthermore, the linear regression analysis method was used to analyze (**Table 4**). Binary logistic regression was used to estimate ORs and 95% confidence intervals (CIs) adjusted for multiple covariates in a different model. In the first model, adjustments were made for age, sex, and BMI. The second model underwent additional modifications to account for education, supplement intake of multivitamin–minerals (vitamins A, D, C, 89, calcium, and omega-3), physical activity, smoking, comorbidity, menopausal status, use of drugs or hormone therapy, and a special diet. The final model additionally incorporated the intake of energy, protein, fiber, calcium, vitamins (D, C, and B9), saturated fatty acids (SFAs), monounsaturated fatty acids (MUFAs), and polyunsaturated fatty acids (PUFAs). In all models, participants in the lowest tertiles of DIL and DII were designated as the reference group. In adjusted models, confounders were used from statistical and conceptual approaches, respectively. In this way, the variables with a *P*-value of < 0.2 were considered as possible confounders and were entered into the logistic regression, and the odds of getting osteoporosis/low BMD were investigated. Furthermore, in the conceptual approach of adjusting confounders in model 3, possible confounders were selected based on clinical concepts and based on past articles and added to other confounders. The data were presented as mean ± SD and OR with 95% CI, and in all results, the significance level was determined as a *p*-value of < 0.05.

## Results

The mean (± SD) age of the study population (33% men) was 43.81 ± 6.97 years. The mean (± SD) BMI was 26.45 ± 3.95 kg/m^2^. Furthermore, the baseline mean ± SD of dietary indices including DII, DIL, DGI, and DGL were 56.51 ± 4.581, 312.94 ± 30.82, 70.2 ± 5.8, and 216.72 ± 28.12, respectively, among all participants in the study. The mean T-score and *Z*-score BMD in total participants were −1.15 and −1.27, respectively.

The baseline characteristics and dietary intakes of the study population based on the tertiles of dietary IL are shown in Table 1. Across tertiles of DIL, the weight, male present, and dietary intakes of energy, carbohydrate, fat, SFA, MUFA, PUFA, cholesterol, phosphorus, iron, sodium, red and processed meat, and refined grains were increased. However, BMD (*T*- and *Z*-scores), physical activity, the percentage of people receiving supplements (multivitamins, vitamin D, B, folate, calcium, iron, and omega-3), and dietary intakes of fruits, vegetables, total dairy, whole grains, nuts and legumes, fish, protein, and antioxidant nutrients and vitamins were decreased across tertiles of DIL. For other variables, there were no significant differences across tertiles of DIL.

**Table 1:** Baseline characteristics among 12,696 participants of Study based on tertiles of dietary insulin load.

**Variables**		**Total population**	**Insulin load**			
			**T1 (*****n*** = **4,232)**	**T2 (*****n*** = **4,232)**	**T3 (*****n*** = **4,232)**	***P*** **-value** ^a^
Age (years)		43.81 (6.97)	43.84 (7.18)	43.61 (6.78)	43.98 (6.93)	0.059
Male, *n* (%)		4,199 (33.1)	874 (20.7)	1231 (29.1)	2094 (49.75)	**< 0.001**
Weight (kg)		72.16 (12.31)	70.41 (11.74)	71.41 (12.27)	74.65 (12.50)	**< 0.001**
BMI (kg/m^2^)		26.45 (3.95)	26.45 (3.96)	26.39 (3.96)	26.51 (3.93)	0.411
BMD femoral (*T*-score)		−1.15 (0.75)	−1.35 (0.99)	**–**1.18 (0.80)	**–**0.97 (0.71)	**< 0.001**
BMD femoral (*Z*-score)		−1.27 (0.71)	−1.39 (0.96)	−1.11 (0.83)	−0.93 (0.69)	**< 0.001**
Physical activity (Met/min/week)		902.71 (905.03)	934.57 (918.99)	913.95 (913.80)	883.74 (913.06)	**0.036**
Osteoporosis, *n* (%)		611 (4.7)	168 (28.0)	204 (34.0)	228 (38.0)	**0.008**
Under a special diet, *n* (%)		2,168 (16.7)	826 (19.5)	718 (17.0)	578 (13.7)	**< 0.001**
Menopausal status (postmenopausal), *n* (%)		1,274 (15.3)	508 (15.5)	467 (15.8)	299 (14.3)	**0.323**
Education	Under diploma	234 (1.8)	73 (1.7)	69 (1.6)	76 (1.8)	**0.008**
	Diploma	521 (4.0)	152 (3.6)	162 (3.8)	194 (4.6)	
	Bachelor's degree	1,758 (13.6)	621 (14.7)	559 (13.2)	536 (12.7)	
	Master's degree	8,324 (64.2)	2,751 (65.0)	2,709 (64.0)	2,720 (64.3)	
	Doctorate and above	2,121 (16.4)	635 (15.0)	733 (17.3)	706 (16.7)	
Current smoker, *n* (%)		799 (6.29)	310 (7.32)	198 (4.67)	291 (6.87)	0.091
Multivitamin intake, *n* (%)		916 (7.1)	330 (7.8)	315 (7.4)	255 (6.0)	**< 0.001**
Vitamin D supplement intake, *n* (%)		2,603 (20.1)	897 (21.2)	892 (21.1)	777 (18.4)	**< 0.001**
Vitamin A supplement intake, *n* (%)		394 (3.0)	130 (3.1)	132 (3.1)	119 (2.8)	0.168
Vitamin C supplement intake, *n* (%)		603 (4.7)	183 (4.3)	227 (5.4)	179 (4.2)	0.065
Vitamin B supplement intake, *n* (%)		573 (4.4)	220 (5.2)	199 (4.7)	145 (3.4)	**< 0.001**
Folate supplement intake, *n* (%)		291 (2.2)	95 (2.2)	109 (2.6)	81 (1.9)	**< 0.001**
Calcium supplement intake, *n* (%)		1,446 (11.2)	495 (11.7)	507 (12.0)	425 (10.0)	**< 0.001**
Iron supplement intake, *n* (%)		2,092 (16.1)	772 (18.2)	732 (17.3)	554 (13.1)	**< 0.001**
Omega 3 supplement intake, *n* (%)		656 (5.1)	243 (5.7)	244 (5.8)	158 (3.7)	**< 0.001**
**Dietary intakes**						
Fruits (g/d)		394.85 (274.58)	441.30 (309.98) 2)	406.47 (267.16)	336.78 (230.4)	**< 0.001**
Vegetables (g/d)		426.32 (339.71)	455.22 (351.60)	425.98 (335.37)	397.77 (329.41)	**< 0.001**
Processed meat (g/d)		17.03 (16.96)	15.50 (14.79)	17.21 (16.43)	18.37 (19.24)	**< 0.001**
Total dairy (g/d)		232.28 (200.67)	268.81 (232.60)	236.13 (195.92)	191.89 (159.23)	**< 0.001**
Legumes (g/d)		14.65 (15.07)	17.82 (18.58)	14.22 (13.54)	11.91 (11.60)	**< 0.001**
Nuts (g/d)		8.18 (10.80)	8.93 (12.28)	8.62 (10.87)	6.98 (8.88)	**< 0.001**
Whole grains (g/d)		99.95 (75.69)	179.11 (73.14)	82.12 (31.26)	38.62 (22.70)	**< 0.001**
Refined grains (g/d)		354.44 (176.10)	208.81 (84.43)	329.11 (99.94)	525.41 (157.63)	**< 0.001**
Fish (g/d)		22.59 (32.78)	26.31 (42.07)	22.68 (29.64)	18.77 (23.38	**< 0.001**
Energy (Kcal/d)		2,017.00 (728.79)	1,353.10 (278.37)	1,884.03 (304.74)	2,813.87 (574.13)	**< 0.001**
Protein (g/d)		61.15 (23.75)	84.19 (21.01)	57.73 (13.69)	41.54 (11.66)	**< 0.001**
Fat (g/d)		51.96 (25.56)	44.40 (21.35)	50.31 (22.71)	61.17 (29.04)	**< 0.001**
Carbohydrate (mg/d)		311.06 (127.94)	189.02 (39.41)	286.77 (39.62)	457.39 (95.32)	**< 0.001**
Cholesterol (mg/d)		159.34 (92.26)	129.13 (67.49)	158.66 (82.19)	190.22 (111.18)	**< 0.001**
PUFA (g/d)		17.23 (11.02)	15.37 (10.36)	16.48 (10.13)	19.85 (11.99)	**< 0.001**
MUFA (g/d)		14.41 (7.33)	12.11 (5.77)	13.96 (6.47)	17.17 (8.55)	**< 0.001**
SFA (g/d)		14.42 (6.72)	12.10 (4.91)	14.19 (5.89)	16.96 (8.07)	**< 0.001**
Calcium (mg/d)		677.41 (355.44)	830.68 (395.83)	674.10 (323.71)	527.44 (267.58)	**< 0.001**
Iron (mg/d)		14.22 (5.91)	8.77 (1.98)	13.04 (2.11)	20.85 (4.64)	**< 0.001**
Phosphorus (mg/d)		1,048.13 (410.37)	751.99 (248.74)	1,009.33 (294.35)	1,383.07 (394.12)	**< 0.001**
Magnasium (mg/d)		326.96 (131.36)	458.96 (109.48)	305.13 (72.43)	216.80 (67.06)	**< 0.001**
Zinc (mg/d)		9.63 (3.74)	13.51 (3.11)	9.02 (1.86)	6.36 (1.68)	**< 0.001**
Sodium (mg/d)		2,103.45 (1,020.77)	1,358.89 (589.54)	1,940.25 (685.35)	3,011.20 (950.67)	**< 0.001**
Vitamin A (mcg/d)		456.49 (315.29)	523.68 (407.96)	453.11 (262.21)	392.68 (233.42)	**< 0.001**
Folate (mcg/d)		269.73 (99.07)	319.66 (112.61)	271.32 (83.91)	218.22 (67.66)	**< 0.001**
Vitamin D (mcg/d)		1.43 (1.87)	1.56 (2.39)	1.42 (1.95)	1.36 (1.69)	**0.006**
Vitamin C (mg/d)		83.97 (54.61)	93.44 (60.34)	85.52 (53.16)	72.95 (47.62)	**< 0.001**
Vitamin B6 (mg/d)		1.50 (0.61)	1.84 (0.67)	1.48 (0.52)	1.18 (0.44)	**< 0.001**
Vitamin B12 (mg/d)		3.35 (2.90)	3.96 (4.07)	3.33 (2.23)	2.76 (1.74)	**< 0.001**
Dietary fibre (g/d)		24.60 (10.74)	35.07 (9.28)	22.76 (6.33)	15.98 (5.71)	**< 0.001**

BMI, body mass index; MET, metabolic equivalent.

Data are presented as mean [standard deviation (SD)] or number (percent).

^a^Obtained from ANOVA or Chi-square test, where appropriate.

Bolded numbers indicate a *p*-value < 0.05.

General characteristics and dietary intake of subjects across tertiles of DGL are presented in Table 2. Compared with those in the lowest tertiles of DGL, subjects in the highest tertile had a higher male present, age, and weight, as well as lower BMD (*T*- and *Z*-scores) and physical activity. In addition, there was a significant difference between the tertiles of DGL in terms of following a special diet, education level, and receiving supplements (multivitamins, vitamin D, B, folate, calcium, iron, and omega-3). No other significant difference was found in other general characteristics across the tertiles of DGL. Furthermore, individuals with the highest tertiles of DGI had a higher intake of energy, carbohydrate, fat, SFA, MUFA, PUFA, cholesterol, phosphorus, iron, sodium, red and processed meat, and refined grains, as well as a lower intake of protein, fiber, calcium, magnesium, zinc, vitamin A, folate, vitamin C, B6, B12, total dairy, whole grains, fish, nuts, legumes, fruits, and vegetables compared with those in the bottom tertiles.

**Table 2:** Baseline characteristics among 12,696 participants of study based on tertiles of dietary glycemic load.

**Variables**		**Total population**	**Glycemic load**			
			**T1 (*****n*** = **4,232)**	**T2 (*****n*** = **4,232)**	**T3 (*****n*** = **4,232)**	***P*** **-value**
Age (years)		43.81 (6.97)	43.92 (7.11)	43.58 (6.84)	43.93 (6.94)	**0.040**
Male, *n* (%)		4,199 (33.1)	937 (22.1)	1,242 (29.3)	2,020 (47.7)	**< 0.001**
Weight (kg)		72.16 (12.31)	70.60 (11.80)	71.41 (12.32)	74.47 (12.46)	**< 0.001**
BMI (kg/m^2^)		26.45 (3.95)	26.46 (3.96)	26.39 (3.99)	26.50 (3.90)	0.456
BMD femoral (*T*-score or *Z*-score)		−1.15 (0.75)	−1.30 (0.90)	−1.12 (0.84)	−0.99 (0.75)	**< 0.001**
BMD femoral (*Z*-score)		−1.27 (0.71)	−1.25 (0.92)	−1.13 (0.85)	−0.90 (0.68)	**< 0.001**
Physical activity (Met/min/week)		902.71 (905.03)	941.08 (909.23)	909.15 (901.21)	871.09 (891.12)	**0.042**
Osteoporosis, *n* (%)		611 (4.7)	180 (30.0)	192 (32.0)	228 (38.0)	**0.038**
Under a special diet, *n* (%)		2,168 (16.7)	812 (19.2)	722 (17.1)	588 (13.9)	**< 0.001**
Menopausal status (postmenopausal), *n* (%)		1,274 (15.3)	497 (15.4)	452 (15.4)	325 (15.0)	**0.896**
Education	Under diploma	234 (1.8)	73 (1.6)	64 (1.5)	81 (1.9)	**0.002**
	Diploma	521 (4.0)	172 (4.1)	150 (3.5)	186 (4.4)	
	Bachelor's degree	1,758 (13.6)	604 (14.3)	557 (13.2)	555 (13.1)	
	Master's degree	8,324 (64.2)	2,771 (65.5)	2,710 (64.0)	2,699 (63.8)	
	Doctorate and above	2,121 (16.4)	612 (14.5)	751 (17.7)	711 (16.8)	
Current smoker, *n* (%)		799 (6.29)	298 (7.04)	305 (7.20)	196 (4.63%)	0.091
Multivitamin intake, *n* (%)		916 (7.1)	320 (7.6)	320 (7.6)	260 (6.1)	**< 0.001**
Vitamin D supplement intake, *n* (%)		2,603 (20.1)	879 (20.8)	889 (21.0)	798 (18.9)	**< 0.001**
Vitamin A supplement intake, *n* (%)		394 (3.0)	133 (3.1)	121 (2.9)	127 (3.0)	0.335
Vitamin C supplement intake, *n* (%)		603 (4.7)	184 (4.3)	221 (5.2)	184 (4.3)	0.201
Vitamin B supplement intake, *n* (%)		573 (4.4)	230 (5.4)	191 (4.5)	143 (3.4)	**< 0.001**
Folate supplement intake, *n* (%)		291 (2.2)	95 (2.2)	102 (2.4)	88 (2.1)	**< 0.001**
Calcium supplement intake, *n* (%)		1,446 (11.2)	471 (11.1)	520 (12.3)	436 (10.3)	**< 0.001**
Iron supplement intake, *n* (%)		2,092 (16.1)	739 (17.5)	728 (17.2)	591 (14.0)	**< 0.001**
Omega 3 supplement intake, *n* (%)		656 (5.1)	241 (5.7)	237 (5.6)	167 (3.9)	**< 0.001**
**Dietary intakes**						
Fruits (g/d)		394.85 (274.58)	483.85 (327.30)	404.44 (251.89)	296.25 (194.69	**< 0.001**
Vegetables (g/d)		426.32 (339.71)	475.30 (360.36)	424.62 (332.27)	379.05 (318.37)	**< 0.001**
Processed meat (g/d)		17.03 (16.96)	15.56 (14.97)	17.03 (16.16)	18.49 (19.33)	**< 0.001**
Total dairy (g/d)		232.28 (200.67)	282.58 (241.38)	232.77 (189.71)	181.48 (146.49)	**< 0.001**
Legumes (g/d)		14.65 (15.07)	18.25 (18.53)	14.11 (13.59)	11.59 (11.43)	**< 0.001**
Nuts (g/d)		8.18 (10.80)	9.47 (12.66)	8.60 (10.81)	6.47 (8.24)	**< 0.001**
Whole grains (g/d)		99.95 (75.69)	172.17 (79.51)	84.00 (38.79)	43.67 (26.94)	**< 0.001**
Refined grains (g/d)		354.44 (176.10)	211.82 (82.51)	329.72 (98.98)	521.78 (165.78)	**< 0.001**
Fish (g/d)		22.59 (32.78)	25.60 (38.89)	22.48 (31.24)	19.69 (26.79)	**< 0.001**
Energy (Kcal/d)		2,017.00 (728.79)	1,356.55 (282.10)	1,882.94 (306.05)	2,811.50 (578.61)	**< 0.001**
Protein (g/d)		61.15 (23.75)	83.73 (21.17)	57.65 (14.22)	42.08 (12.45)	**< 0.001**
Fat (g/d)		51.96 (25.56)	44.96 (21.87)	49.95 (22.75)	60.98 (28.80)	**< 0.001**
Carbohydrate (mg/d)		311.06 (127.94)	187.78 (37.25)	287.23 (36.23)	458.17 (94.86)	**< 0.001**
Cholesterol (mg/d)		159.34 (92.26)	131.16 (70.43)	156.73 (81.54)	190.13 (110.36)	**< 0.001**
PUFA (g/d)		17.23 (11.02)	15.65 (10.56)	16.38 (10.13)	19.67 (11.88)	**< 0.001**
MUFA (g/d)		14.41 (7.33)	12.28 (5.91)	13.82 (6.44)	17.14 (8.52)	**< 0.001**
SFA (g/d)		14.42 (6.72)	12.19 (5.11)	14.08 (5.89)	16.97 (7.96)	**< 0.001**
Calcium (mg/d)		677.41 (355.44)	848.96 (394.00)	667.68 (309.99)	515.57 (268.35)	**< 0.001**
Iron (mg/d)		14.22 (5.91)	8.86 (2.06)	13.08 (2.29)	20.72 (4.82)	**< 0.001**
Phosphorus (mg/d)		1,048.13 (410.37)	746.25 (248.61)	1,004.12 (282.95)	1,394.01 (388.16)	**< 0.001**
Magnasium (mg/d)		326.96 (131.36)	460.09 (109.86)	305.70 (69.76)	215.09 (64.35)	**< 0.001**
Zinc (mg/d)		9.63 (3.74)	13.42 (3.20)	9.02 (1.96)	6.46 (1.77)	**< 0.001**
Sodium (mg/d)		2,103.45 (1,020.77)	1,354.50 (528.43)	1,947.57 (681.28)	3,008.27 (989.32)	**< 0.001**
Vitamin A (mcg/d)		456.49 (315.29)	540.59 (410.64)	450.50 (254.59)	378.39 (227.20)	**< 0.001**
Folate (mcg/d)		269.73 (99.07)	330.87 (110.56)	268.39 (76.88)	209.94 (63.23)	**< 0.001**
Vitamin D (mcg/d)		1.43 (1.87)	1.48 (2.00)	1.50 (2.25)	1.43 (1.84)	0.089
Vitamin C (mg/d)		83.97 (54.61)	100.44 (63.51)	85.18 (51.99)	66.29 (40.34)	**< 0.001**
Vitamin B6 (mg/d)		1.50 (0.61)	1.90 (0.66)	1.47 (0.48)	1.13 (0.39)	**< 0.001**
Vitamin B12 (mg/d)		3.35 (2.90)	3.97 (4.03)	3.29 (2.20)	2.79 (1.89)	**< 0.001**
Dietary fibre (g/d)		24.60 (10.74)	35.55 (9.12)	22.85 (5.64)	15.41 (4.88)	**< 0.001**

Bolded numbers indicate a *p*-value < 0.05.

The ORs and 95% CIs for osteoporosis subjects based on the tertiles of available glycemic and insulin indices are reported in Table 3.

**Table 3:** Odds ratio (OR) and 95% confidence interval (CI) for osteoporosis based on dietary insulin and glycemic scores among participants.

	**Tertiles of scores**			***P* for trend**
	**T1 (*****n*** = **4,232)**	**T2 (*****n*** = **4,232)**	**T3 (*****n*** = **4,232)**	
**Glycemic Index**				
**Mean (SD) score**	64.70 (3.36)	69.72 (1.07)	76.18 (4.75)	
Crude model	1.00 (Ref)	2.28 (1.23–3.21)	2.10 (1.14–2.90)	**0.021**
Model 1^*^	1.00 (Ref)	2.18 (0.95–3.99)	1.74 (0.76–2.95)	**0.019**
Model 2^‡^	1.00 (Ref)	1.85 (0.37–3.08)	1.72 (0.17–2.81)	**0.043**
Model 3^¥^	1.00 (Ref)	1.82 (0.66–3.65)	1.78 (1.90–3.52)	**0.001**
**Glycemic load**				
**Mean (SD) score**	129.56 (25.14)	202.09 (21.25)	321.72 (39.05)	
Crude model	1.00 (Ref)	1.31 (1.06–1.62)	1.36 (1.10–1.68)	**0.004**
Model 1^*^	1.00 (Ref)	1.19 (0.96–1.48)	0.95 (0.76–1.19)	0.842
Model 2^‡^	1.00 (Ref)	1.22 (0.98–1.52)	0.97 (0.78–1.22)	0.998
Model 3^¥^	1.00 (Ref)	1.37 (1.05–1.79)	1.46 (1.17–2.02)	**0.035**
**Insulin index**				
**Mean (SD) score**	48.59 (4.93)	57.19 (1.65)	63.77 (3.69)	
Crude model	1.00 (Ref)	1.06 (0.86–1.31)	1.10 (0.89–1.36)	0.371
Model 1^*^	1.00 (Ref)	1.00 (0.81–1.25)	1.03 (0.88–1.28)	0.942
Model 2^‡^	1.00 (Ref)	1.01 (0.81–1.26)	0.98 (0.79–1.11)	0.250
Model 3^¥^	1.00 (Ref)	1.01 (0.78–1.31)	1.16 (0.93–1.27)	0.642
**Insulin load**				
**Mean (SD) score**	281.33 (35.22)	298.45 (26.93)	359.06 (23.25)	
Crude model	1.00 (Ref)	1.17 (0.95–1.44)	1.46 (1.17–1.82)	**0.001**
Model 1^*^	1.00 (Ref)	1.11 (0.89–1.32)	1.39 (1.05–1.77)	**0.037**
Model 2^‡^	1.00 (Ref)	1.06 (0.85–1.31)	0.98 (0.78–1.24)	0.512
Model 3^¥^	1.00 (Ref)	1.22 (0.91–1.59)	1.55 (1.09–2.31)	0.104

Binary logistic regression was used to obtain OR and 95% CI.

^*^Model 1: adjusted for age; sex; and BMI.

^‡^Model 2: Model 1 + education; supplement intake of multivitamin-mineral, vitamin A, D, C, B9, calcium, omega 3; physical activity; smoking; Comorbidity; menopausal status, use of drug or hormone therapy, under a special diet.

^¥^Model 3: Model 2 + intake of energy; protein; fiber; phosphorus, calcium; vitamin D, C, and B9; SFA; MUFA; PUFA.

Bolded numbers indicate a *p*-value < 0.05.

In the crude model, the DGI and DGL were directly associated with the odds of osteoporosis, with an OR of 2.10 and 1.36 for the highest tertile, respectively, as compared to the lowest tertile (*P* < 0.05 for trend). Furthermore, after we adjusted for age, sex, BMI, education, supplement intake of multivitamin–mineral, vitamin A, D, C, B9, calcium, omega-3, physical activity, smoking, comorbidity, menopausal status, use of drug or hormone therapy, under a special diet, as well as intake of energy, protein, fiber, phosphorus, calcium, vitamin D, C, B9, SFA, MUFA, and PUFA (in the fully adjusted model), in the highest vs. lowest tertile of GI and GL, the increase odds of osteoporosis remained significant (OR = 1.78, 95% CI: 1.90–3.52; *P* trend = 0.001 for trend and OR = 1.46, 95% CI: 1.17–2.02; *P* trend = 0.035 for trend, respectively).

On the other hand, in the first tertile compared to the last tertile, although an increase in the DIL score was associated with higher odds of osteoporosis in both the crude and first models (OR = 1.46, 95% CI: 1.17–1.82; *P* trend = 0.001 for trend and OR = 1.39, 95% CI: 1.05–1.77; *P* trend = 0.037 for trend, respectively), no significant relationship between the DII and the odds of this disease was observed in these two models (OR = 1.10, 95% CI: 0.89–1.36; *P* trend = 0.371 for trend and OR = 1.03, 95% CI: 0.88–1.28; *P* trend = 0.942 for trend, respectively). In addition, after adjusting for possible confounders in the final adjusted model, no significant relationship between dietary insulin indices (DII and DIL) and the odds of osteoporosis was observed (OR = 1.16, 95% CI: 0.93–1.27; *P* trend = 0.642 for trend and OR = 1.55, 95% CI: 1.09–2.31; *P* trend = 0.104 for trend, respectively) (Table 3).

The linear relationship between the T-score and the scores examined in the study is also shown in Table 4. However, no significant linear relationship between *T*-score and different scores was observed.

**Table 4:** Linear relationship between *T*-score and dietary insulin and glycemic scores among 12,696 participants.

**Scores**	**Total** ***T*****-score**		***P*-Value**
	**SE**	β	
Glycemic index^*^	0	−0.001	0.055
Glycemic load^*^	0.02	−0.018	0.39
Insulin index^*^	0.012	−0.018	0.153
Insulin load^*^	0.676	0.185	0.787

^*^Model 1: adjusted for age; sex; and BMI, education; supplement intake of multivitamin-mineral, vitamin A, D, C, B9, calcium, omega 3; physical activity; smoking; Comorbidity; menopausal status, use of drug or hormone therapy, under a special diet, intake of energy; protein; fiber; calcium; vitamin D, C, and B9; SFA; MUFA; PUFA.

## Discussion

This study indicates a significant association between high DGI/DGL and an increased odds of osteoporosis. According to our findings, being in the last tertile of the GI and GL is associated with 78 and 46% higher odds of osteoporosis, respectively.

Our findings are consistent with some previous studies, including one that demonstrated a link between high DGI and DGL with an elevated risk of fracture in an elderly Mediterranean population (23) and another study that found an association between BMD abnormality and DGI in postmenopausal women (24). In addition, one study has shown that increased glycemic variability in patients with T2DM is associated with osteoporosis (37).

Evidence shows that high DGI and DGL diets may increase oxidative stress and inflammation (38–40), which can impair bone health by stimulating osteoclastogenesis and bone resorption (41) and inhibiting osteoblast function (42). Moreover, several cytokines may contribute to osteoporosis pathogenesis, notably interleukin (IL)-6 (43), tumor necrosis factor (TNF)-α (44), and IL-1 (45). The direct and indirect effects of IL-6 on osteoclastic processes have been extensively studied (46–48). It has been shown that the inhibition of IL-6 receptor signaling inhibits the formation of osteoclasts both *in vitro* and *in vivo* (49). IL-6 can also inhibit osteoclast differentiation directly by acting on osteoclast progenitors (47). Furthermore, evidence suggests that TNF-α promotes bone resorption *in vitro* (44). TNF-α acts directly on surface receptors and induces the differentiation of osteoclasts (50). It also inhibits osteoblast function and bone formation by inhibiting insulin-like growth factor (IGF)-I expression and RUNX2 expression in osteoblast precursor cells (51, 52). Osteoclastogenesis may be directly stimulated by IL-1, specifically IL-1b, or indirectly induced by TNF-α (53). In fact, IL-1 stimulates osteoclast differentiation in bone marrow-derived macrophages by signaling through IL-1/IL-1R1 (54). Furthermore, high-glycemic foods tend to be more refined and processed than low-glycemic foods, which can lead to nutritional deficiencies (55). Similarly, our study found that individuals with a high GL tended to consume more processed meat and refined grains while consuming fewer fruits, vegetables, nuts, fish, whole grains, and dairy products. Hence, consuming a diet predominantly composed of high-glycemic index foods may result in inadequate intake of key nutrients required for optimal bone health, such as calcium, vitamin D, magnesium, and vitamin K. These nutrients play critical roles in bone formation, mineralization, and maintaining bone density (56). In addition, meat and grains typically impart a high dietary acid load, whereas fruits and vegetables provide an alkaline load (57–59). In the presence of a slight drop in the pH of the extracellular fluid, osteoblast activity will be suppressed, and matrix protein gene expression and alkaline phosphatase activity will be decreased (60). Furthermore, low-grade metabolic acidosis decreases bone calcium deposits by increasing urinary calcium excretion (61, 62). Finally, hyperglycemia promotes the production of advanced glycation end products (AGEs), which increase the cross-linking of collagens and the fragility of human bones (63).

Intriguingly, our study revealed a lack of significant correlation between DII and DIL and the risk of osteoporosis. This finding is consistent with that of Nouri et al., who found no correlation between DII and DIL and lumbar/femoral BMD in postmenopausal women (24). Previous studies have reported conflicting results regarding the impact of hyperinsulinemia on BMD. Some studies suggest that hyperinsulinemia increases BMD (9, 64, 65), while others observe a decrease (66, 67). In a recent study, a significant relationship between decreased BMD or osteoporosis with increased homeostatic model assessment for insulin resistance (HOMA-IR) was reported (68). By contrast, in a study by Napoli et al. (69), greater insulin resistance (IR) was associated with higher BMD in non-diabetic elderly subjects, suggesting that IR may affect fracture risk possibly through effects on bone quality. In line with this observation, patients with T2DM have an increased fracture risk despite normal or even slightly elevated BMD and frequently show impaired bone microstructure. However, consistent evidence that IR is associated with increased fracture risk after adjusting for BMI, and BMD was not observed in the aforementioned study. The lack of significant association in our study may be due to variations in study populations, methodologies, or other confounding factors that were not taken into account. So, it seems that the role of the dietary insulin response in osteoporosis development requires further investigation. In addition, the low prevalence of postmenopausal women (who have a low risk of osteoporosis) in the study population can also justify the lack of significant results and differences with other studies.

This cross-sectional study provides valuable insight into the association between high DGI/DGL and osteoporosis risk. Our study's strengths include its relatively large sample size, which enhances the statistical power and generalizability of the findings to the target population. Additionally, using validated assessment tools for dietary intake and osteoporosis risk factors adds credibility to our data. Including diverse participants from different ethnographic and geographic backgrounds also strengthens the external validity of our findings.

However, we must acknowledge that this study has some limitations. First of all, due to the cross-sectional design, we are unable to establish causality between high DGI/DGL and osteoporosis risk. The temporal sequence of events cannot be determined, and the possibility of reverse causation remains. Longitudinal studies would be valuable in elucidating the cause-and-effect relationship. The second limitation is that dietary assessments in cross-sectional studies are subject to recall bias and rely heavily on self-reported information. Participants' memory, perception, and social desirability bias may influence dietary information accuracy. Despite efforts to minimize these biases through validated questionnaires and recruiting teachers as participants, the potential for measurement error cannot be eliminated entirely. Failure to report weather-related data as one of the influencing factors in the incidence of osteoporosis was one of the limitations of our study. Additionally, as with any observational study, confounding variables can influence the association between high DGI/DGL/DII/DIL and osteoporosis risk. While we adjusted for a number of potential confounders, residual confounding may arise from unmeasured or unknown factors. As a final point, our findings may be confined to the particular population under study.

In conclusion, our study provides evidence of a significant association between high DGI and DGL and increased osteoporosis prevalence. However, due to the limited literature available and the complexity of the underlying mechanisms, further research is needed to validate these findings and elucidate the precise pathways involved. Additionally, the lack of a significant relationship between DII/DIL and osteoporosis prevalence in our study warrants further exploration. By addressing these knowledge gaps, we can better understand the impact of dietary factors on osteoporosis and potentially develop targeted interventions to mitigate the risk.

## Data Availability

The raw data supporting the conclusions of this article will be made available by the authors, without undue reservation.
